# Metabolomic changes in animal models of depression: a systematic analysis

**DOI:** 10.1038/s41380-021-01269-w

**Published:** 2021-09-01

**Authors:** Juncai Pu, Yiyun Liu, Siwen Gui, Lu Tian, Yue Yu, Xuemian Song, Xiaogang Zhong, Xiaopeng Chen, Weiyi Chen, Peng Zheng, Hanping Zhang, Xue Gong, Lanxiang Liu, Jing Wu, Haiyang Wang, Peng Xie

**Affiliations:** 1 NHC Key Laboratory of Diagnosis and Treatment on Brain Functional Diseases, The First Affiliated Hospital of Chongqing Medical University, Chongqing, China; 2 Department of Neurology, The First Affiliated Hospital of Chongqing Medical University, Chongqing, China; 3 Department of Health Sciences Research, Mayo Clinic, Rochester, MN USA

**Keywords:** Molecular biology, Depression

## Abstract

Extensive research has been carried out on the metabolomic changes in animal models of depression; however, there is no general agreement about which metabolites exhibit constant changes. Therefore, the aim of this study was to identify consistently altered metabolites in large-scale metabolomics studies of depression models. We performed vote counting analyses to identify consistently upregulated or downregulated metabolites in the brain, blood, and urine of animal models of depression based on 3743 differential metabolites from 241 animal metabolomics studies. We found that serotonin, dopamine, gamma-aminobutyric acid, norepinephrine, N-acetyl-L-aspartic acid, anandamide, and tryptophan were downregulated in the brain, while kynurenine, myo-inositol, hydroxykynurenine, and the kynurenine to tryptophan ratio were upregulated. Regarding blood metabolites, tryptophan, leucine, tyrosine, valine, trimethylamine N-oxide, proline, oleamide, pyruvic acid, and serotonin were downregulated, while N-acetyl glycoprotein, corticosterone, and glutamine were upregulated. Moreover, citric acid, oxoglutaric acid, proline, tryptophan, creatine, betaine, L-dopa, palmitic acid, and pimelic acid were downregulated, and hippuric acid was upregulated in urine. We also identified consistently altered metabolites in the hippocampus, prefrontal cortex, serum, and plasma. These findings suggested that metabolomic changes in depression models are characterized by decreased neurotransmitter and increased kynurenine metabolite levels in the brain, decreased amino acid and increased corticosterone levels in blood, and imbalanced energy metabolism and microbial metabolites in urine. This study contributes to existing knowledge of metabolomic changes in depression and revealed that the reproducibility of candidate metabolites was inadequate in previous studies.

## Introduction

Depression is a mental illness that severely impairs the social function of patients. The lifetime prevalence of depression is ~6.8 to 20.6% [1, 2]. In addition, depression severely impairs the quality of life of patients and has become one of the primary diseases leading to mental disability [3]. Despite a large body of evidence supporting the pathophysiology of depression, the underlying molecular mechanisms that mediate its onset remain unclear. Depression has complex and diverse causal factors, a lack of clear pathological alterations or risk genes, and a high degree of heterogeneity in clinical presentation [4, 5].

Metabolites are products of upstream gene and protein regulatory networks that are involved in a variety of physiological and pathological conditions [6, 7]. Metabolomics techniques have been rapidly developed in the last two decades [8]. Because of the important physiological role of metabolites, these metabolomics techniques have been widely used to identify biochemical disturbances in diseases [9]. To date, hundreds of metabolomics studies have been conducted to investigate the metabolite alterations in animal models of depression, which expanded the knowledge of the physiopathology of depression [10–12]. Moreover, supplementation with certain metabolites could exert antidepressant effects, suggesting that metabolomics techniques are potential strategies for screening and developing new antidepressants [13–15].

Although extensive research has been carried out on the metabolomic changes in animal models of depression, there are concerns regarding the generalizability of these metabolomics studies, as their results are not consistent [16]. To our knowledge, no previous study has assessed the reproducibility of metabolomics studies, and there is no general agreement about which metabolites show constant changes in animal models of depression. Therefore, the aim of this study was to perform a systematic analysis to identify consistently altered metabolites in animal models of depression by integrating the totality of evidence from these large-scale metabolomics studies. Using a knowledgebase-driven approach, we performed vote counting analyses to identify consistently upregulated or downregulated metabolites in the brain, blood, and urine in animal models of depression.

## Materials and methods

### Data source

Candidate metabolites were derived from our metabolite database of depression called the Metabolite Network of Depression Database (MENDA) (http://menda.cqmu.edu.cn:8080/index.php). Briefly, this database included 464 clinical and preclinical studies that investigated metabolite changes in depression and its treatment using metabolomics and magnetic resonance spectroscopy (MRS) techniques as of March 20, 2018 [17]. The search strategy and study selection criteria can be found in a previously published article [17]. We then manually curated 5675 differential metabolites from these studies. The ratios of two metabolites were also included in this database.

In this study, we updated this database up to April 1, 2021, which nearly doubled the numbers of included studies and metabolites. After researching PubMed, the Cochrane Library, Embase, Web of Science, and PsycINFO, we cumulatively reviewed 2177 full-text articles and excluded 1409 studies (Supplementary Data 1). Finally, we included 768 clinical and preclinical metabolomics and MRS studies that investigated metabolic alterations in depression and its treatment. We did not exclude studies that none of the metabolites tested reached significant levels. A total of 12,097 differential metabolites were manually obtained from these studies based on the significance levels reported in the original studies. We did not use uniform statistical criteria for data reanalysis because only 3% of these studies provided raw data. In addition, we used the Human Metabolome Database (HMDB) [18], PubChem [19], and Kyoto Encyclopedia of Genes and Genomes (KEGG) [20] databases to standardize the synonyms of candidate metabolites.

### Data selection

For further data analysis, we selected curated data according to the following criteria. For study type, we included studies that compared metabolite levels between rodent models of depression and controls and excluded intervention studies that investigated metabolic changes associated with antidepressant treatment. Nonhuman primates and cell studies were excluded. For metabolite detection methods, we included non-targeted and targeted metabolomics studies and excluded in vivo MRS studies. In addition, we excluded metabolites with unknown regulation directions.

### Data analysis strategy

For the quantitative analysis, we used the following analytical strategy. In the main analysis, we analyzed metabolite alterations in brain, blood (plasma and serum), and urine samples. To further analyze the metabolite alterations in different tissues, secondary analyses were performed in the hippocampus, prefrontal cortex, serum, and plasma. Other brain or peripheral tissues were not included because of the small amount of data. In addition, we also explored metabolite alterations in the chronic mild stress (CMS) model, the most commonly used model of depression. The quality of reporting of the included studies was evaluated according to the updated STAIR recommendations [21].

### Statistical analysis

Merging raw data, mean values, or *p* values are the optimal strategies for merging metabolomics data [22, 23]. However, a lack of raw data, mean values, or fold changes for each metabolite in most of these metabolomics studies prevented us from conducting a meta-analysis. Alternatively, we used the vote counting process to analyze whether metabolites were consistently upregulated or downregulated across studies. The vote counting method can enrich for candidate molecules that are likely to be confirmed by independent testing [24]. During the vote counting process, each metabolite was noted as “1” or “−1” when it was reported as significantly upregulated or downregulated in each study, respectively. Where samples were taken from different brain regions in one research, candidate metabolites were considered to originate from independent studies and were counted separately. The vote counting statistic (VCS) for each metabolite was then calculated by summing the scores [25]. Larger or smaller VCS values indicated that more studies reported the metabolite as significantly upregulated or downregulated, respectively.

To analyze whether the upregulation or downregulation of each metabolite was statistically significant, we used a binomial distribution to determine whether the positive significant findings were attributable to chance, with the assumption that each candidate metabolite was upregulated or downregulated in each study with a probability of 50% [25]. The binomial tests were implemented in R (v 4.0.4, https://www.r-project.org/) with the function binom.test. The one-tailed *p* values were calculated for candidate metabolites that were reported in more than three data sets, and the minimum *p* value for fewer data sets was 0.1. Differences with *p* < 0.05 were considered to be significant.

## Results

### Data sets

Through screening of the MENDA database, 8354 differential metabolites from 527 studies were excluded from the study; see Supplementary Table 1 and Supplementary Data 2 for the reasons for exclusion. A total of 3743 differential metabolites from 241 metabolomics studies in animal models of depression were included in the analysis. The full information of included studies (title, study design, category of depression model, organism, tissue, platform, criteria for depression, sample size, original data availability, and citation) is provided in Supplementary Data 3. The full information of candidate metabolites (metabolite name, HMDB ID, KEGG ID, PubChem ID, comparison group, tissue, organism, category of depression model, platform, and regulation direction) is presented in Supplementary Data 4.

**Fig. 1:**
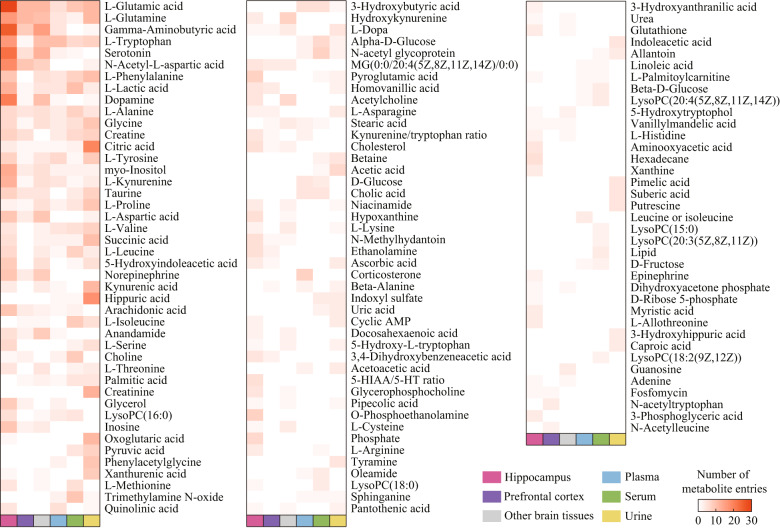
Distribution of metabolites in brain, blood, and urine samples of depression models. Darker colors in the boxes represent larger values. Only metabolites that were reported as dysregulated in more than three studies for any type of sample are shown. *5-HIAA* 5-hydroxyindoleacetic acid, *5-HT* serotonin, *AMP* adenosine monophosphate, *LysoPC* lysophosphatidylcholine, *MG* monoacylglycerol.

The characteristics of the included studies and metabolites are summarized in Supplementary Table 2. Briefly, brain tissues were used in 151 studies, with 2119 differential metabolites. The hippocampus (95 studies, 1119 differential metabolites) and prefrontal cortex (46 studies, 460 differential metabolites) were the most widely used brain tissues. Regarding peripheral tissues, plasma (53 studies, 415 differential metabolites) was the most commonly used sample type, followed by serum (43 studies, 572 differential metabolites), and urine (37 studies, 637 differential metabolites). Eighty-seven unique metabolites (or metabolite ratios) in the brain, 58 in blood, and 45 in urine were reported as dysregulated in at least four studies (Fig. 1). The CMS model was the most widely used animal model (140 studies, 1955 differential metabolites). The overall quality of reporting is presented in Supplementary Data 5, and few studies have met some of these criteria (e.g., sample size calculation and randomization).

### Metabolites altered in the brain

**Fig. 2:**
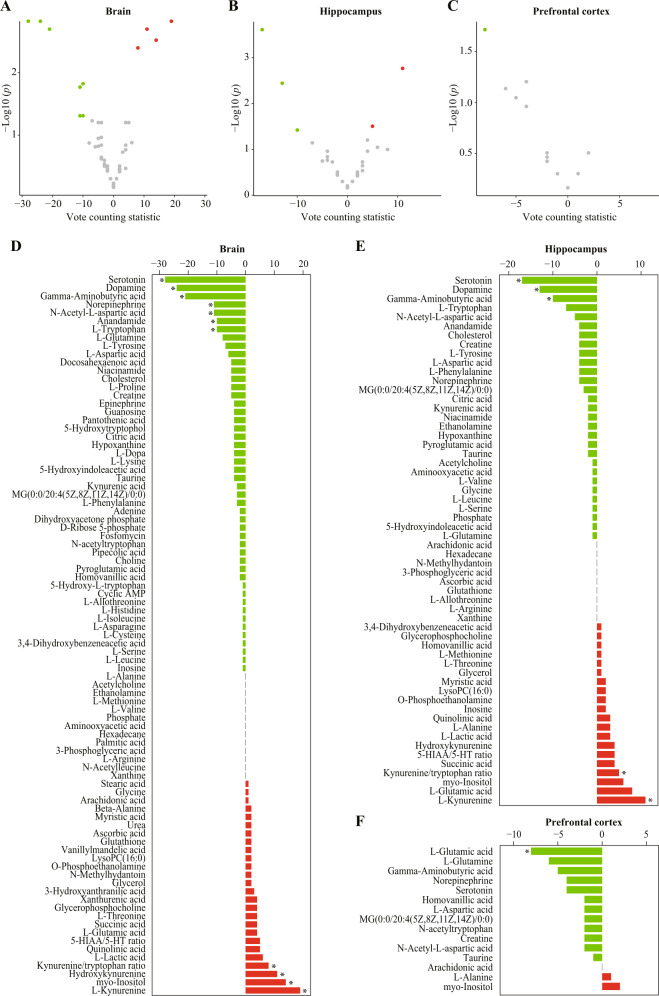
Vote counting results for the brain. **A**–**C** Volcano plots of candidate metabolites in the brain (**A**), hippocampus (**B**), and prefrontal cortex (**C**). Nodes represent candidate metabolites, the *x*-axis shows the vote counting statistic, and the *y*-axis shows the −log10 (*p* value). Red, green, and gray nodes denote consistently upregulated, downregulated, or unaltered metabolites, respectively. **D**–**F** Bar plots of candidate metabolites in the brain (**D**), hippocampus (**E**), and prefrontal cortex (**F**). Red and green bars denote the vote counting statistic for each candidate metabolite. An asterisk (*) indicates *p* < 0.05. *5-HIAA* 5-hydroxyindoleacetic acid, *5-HT* serotonin, *AMP* adenosine monophosphate, *LysoPC* lysophosphatidylcholine, *MG* monoacylglycerol.

We assessed which metabolites were consistently dysregulated in the brain. Volcano plots of candidate metabolites in the brain, hippocampus, and prefrontal cortex are presented in Fig. 2A–C. Among the 85 candidate metabolites and two metabolite ratios in the brain, serotonin (VCS = −28, *p* < 0.001), dopamine (VCS = −24, *p* < 0.001), gamma-aminobutyric acid (GABA, VCS = −21, *p* = 0.002), norepinephrine (VCS = −11, *p* = 0.017), N-acetyl-L-aspartic acid (VCS = −11, *p* = 0.049), anandamide (VCS = −10, *p* = 0.015), and tryptophan (VCS = −10, *p* = 0.049) were downregulated, while kynurenine (VCS = 19, *p* < 0.001), myo-inositol (VCS = 14, *p* = 0.003), hydroxykynurenine (VCS = 11, *p* = 0.002), and the kynurenine to tryptophan ratio (VCS = 8, *p* = 0.004) were upregulated (Fig. 2D and Supplementary Table 3).

Among the 56 candidate metabolites and two metabolite ratios in the hippocampus, serotonin (VCS = −17, *p* < 0.001), dopamine (VCS = −13, *p* = 0.004), and GABA (VCS = −10, *p* = 0.038) were downregulated, and kynurenine (VCS = 11, *p* = 0.002) and the kynurenine to tryptophan ratio (VCS = 5, *p* = 0.031) were upregulated (Fig. 2E and Supplementary Table 4). Among the 15 candidate metabolites in the prefrontal cortex, only glutamic acid was dysregulated (VCS = −8, *p* = 0.019; Fig. 2F and Supplementary Table 5).

### Metabolites altered in blood

**Fig. 3:**
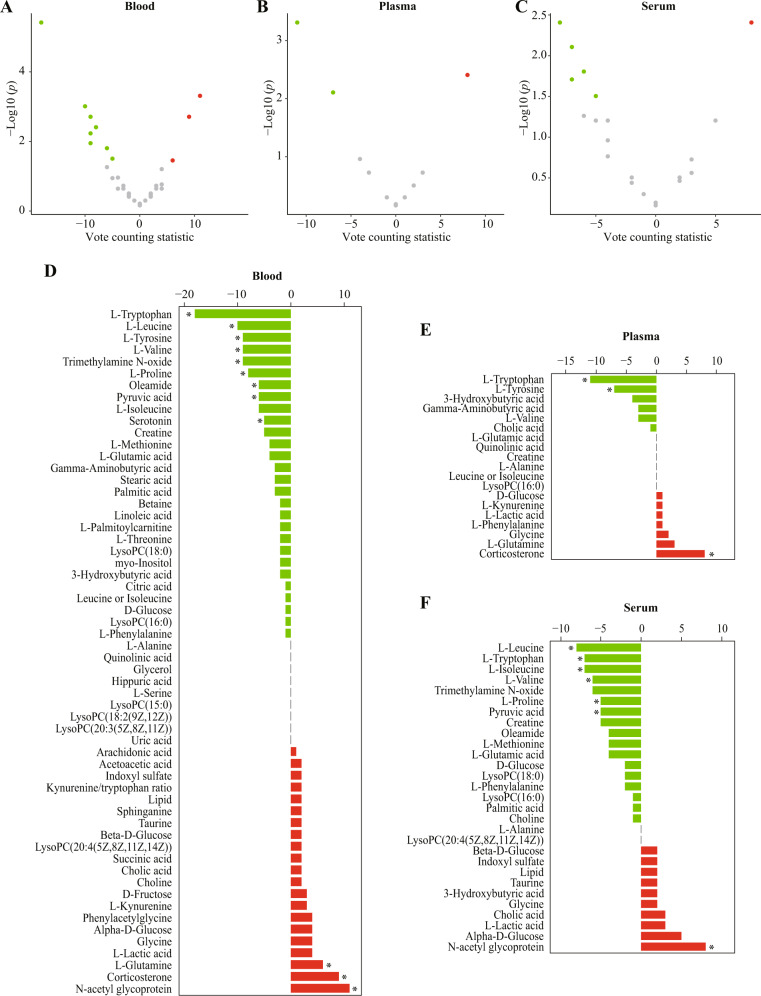
Vote counting results for blood. **A**–**C** Volcano plots of candidate metabolites in blood (**A**), plasma (**B**), and serum (**C**). Nodes represent candidate metabolites, the *x*-axis shows the vote counting statistic, and the *y*-axis shows the −log10 (*p* value). Red, green, and gray nodes denote consistently upregulated, downregulated, or unaltered metabolites, respectively. **D**–**F** Bar plots of candidate metabolites in blood (**D**), plasma (**E**), and serum (**F**). Red and green bars denote the vote counting statistic for each candidate metabolite. An asterisk (*) indicates *p* < 0.05. *LysoPC* lysophosphatidylcholine.

We further analyzed the metabolite alterations in blood. Volcano plots of candidate metabolites in blood, plasma, and serum are presented in Fig. 3A–C. Fifty-seven candidate metabolites and one metabolite ratio in the blood were introduced in the vote counting procedure, and the results revealed that tryptophan (VCS = −18, *p* < 0.001), leucine (VCS = −10, *p* = 0.001), tyrosine (VCS = −9, *p* = 0.002), valine (VCS = −9, *p* = 0.006), trimethylamine N-oxide (VCS = −9, *p* = 0.011), proline (VCS = −8, *p* = 0.004), oleamide (VCS = −6, *p* = 0.016), pyruvic acid (VCS = −6, *p* = 0.016), and serotonin (VCS = −5, *p* = 0.031) were downregulated, while N-acetyl glycoprotein (VCS = 11, *p* < 0.001), corticosterone (VCS = 9, *p* = 0.002), and glutamine (VCS = 6, *p* = 0.035) were upregulated (Fig. 3D and Supplementary Table 6).

Further analysis showed that three and seven candidate metabolites were consistently dysregulated in plasma and serum samples, respectively (Fig. 3E, F). Tryptophan (VCS = −11, *p* < 0.001), tyrosine (VCS = −7, *p* = 0.008), and corticosterone (VCS = 8, *p* = 0.004) were dysregulated in plasma samples (Supplementary Table 7). Leucine (VCS = −8, *p* = 0.004), tryptophan (VCS = −7, *p* = 0.008), isoleucine (VCS = −7, *p* = 0.020), valine (VCS = −6, *p* = 0.016), proline (VCS = −5, *p* = 0.031), pyruvic acid (VCS = −5, *p* = 0.031), and N-acetyl glycoprotein (VCS = 8, *p* = 0.004) were dysregulated in serum samples (Supplementary Table 8).

### Metabolites altered in urine

**Fig. 4:**
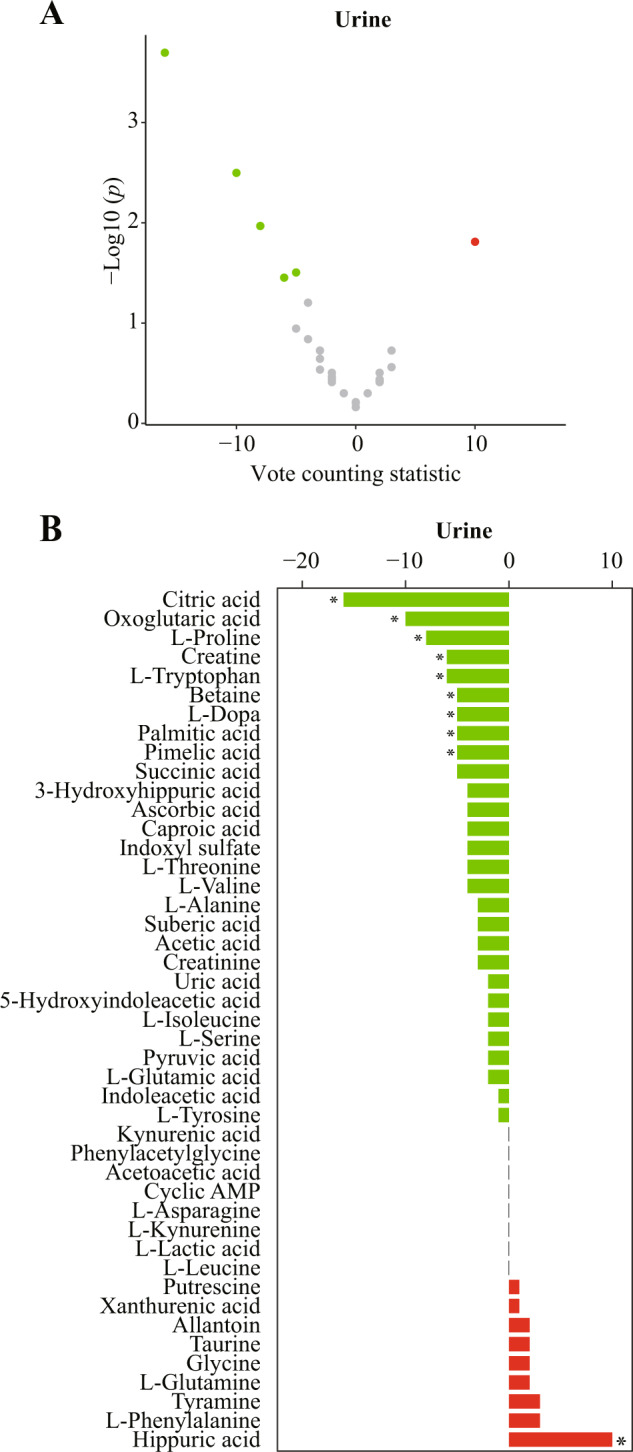
Vote counting results in urine. **A** Volcano plots of candidate metabolites. Nodes represent candidate metabolites, the *x*-axis shows the vote counting statistic, and the *y*-axis shows the −log10 (*p* value). Red, green, and gray nodes denote consistently upregulated, downregulated, or unaltered metabolites, respectively. **B** Bar plots of candidate metabolites. Red and green bars denote the vote counting statistic for each candidate metabolite. An asterisk (*) indicates a *p* < 0.05. *AMP* adenosine monophosphate.

The vote counting procedure revealed that 10 of the 45 candidate metabolites in urine were consistently dysregulated (Fig. 4A). Citric acid (VCS = −16, *p* < 0.001), oxoglutaric acid (VCS = −10, *p* = 0.003), proline (VCS = −8, *p* = 0.011), tryptophan (VCS = −6, *p* = 0.035), creatine (VCS = −6, *p* = 0.035), betaine (VCS = −5, *p* = 0.031), L-dopa (VCS = −5, *p* = 0.031), palmitic acid (VCS = −5, *p* = 0.031), and pimelic acid (VCS = −5, *p* = 0.031) were downregulated, and hippuric acid (VCS = 10, *p* = 0.015) was upregulated (Fig. 4B and Supplementary Table 9).

### Metabolites altered in the CMS model

**Fig. 5:**
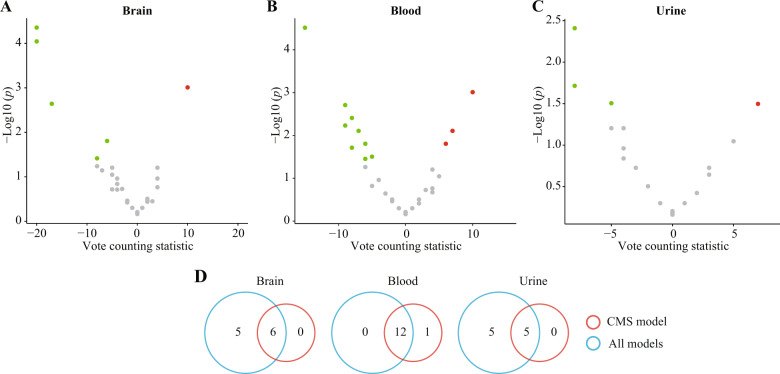
Vote counting results in the chronic mild stress (CMS) model. **A**–**C** Volcano plots of candidate metabolites in the brain (**A**), blood (**B**), and urine (**C**) of the CMS model. Nodes represent candidate metabolites, the *x*-axis shows the vote counting statistic, and the *y*-axis shows the −log10 (*p* value). Red, green, and gray nodes denote consistently upregulated, downregulated, or unaltered metabolites, respectively. **D** Venn diagram displaying the shared consistently dysregulated metabolites in all models and the CMS model.

To determine whether the vote counting results were influenced by types of models, we investigated the altered metabolites in the CMS model. Six, 13, and five metabolites were consistently dysregulated in brain, blood, and urine samples, respectively (Fig. 5A–C; Supplementary Tables 10–12). All but one of these consistently altered metabolites were shared among the results of all models (Fig. 5D). Regarding other types of models, the vast majority of candidate metabolites were reported in less than three data sets, leaving insufficient data for the vote counting procedure.

## Discussion

This study is the first comprehensive investigation of metabolomic changes in animal models of depression that provided a large evidence synthesis of 241 studies. Consistently dysregulated metabolites in brain, blood, and urine samples across these studies were identified from 3743 differential metabolites using a vote counting approach. We found that decreased neurotransmitter levels and increased kynurenine metabolite levels were the main metabolite signatures in the brains of depression models. Blood was characterized by decreased amino acid concentrations and increased corticosterone levels. We also provided evidence of imbalanced energy metabolism and microbial metabolites in urine.

The main results obtained from this study, contrary to our expectations, revealed the inadequate reproducibility of metabolomics studies. Although nearly 300 metabolomics studies were examined, we only identified approximately 10 consistently altered metabolites in brain, blood, and urine samples. This may have occurred because many factors may influence the identification of differential metabolites. Sample preparation, metabolomics experiments, and data analysis are decisive factors that affect the reproducibility of methods [26, 27]. Indeed, any change in parameter settings in these processes can have substantial effects on the compound identification and quantification results [28]. Regarding the issue of result reproducibility, the heterogeneity of depression also needs to be considered. Our previous animal study found that different stress paradigms would lead to diverse patterns of metabolic changes in the hippocampus [29]. Clinical studies also revealed sex and age differences in the plasma metabolome signatures of MDD patients [30, 31]. Despite these concerns, our systematic analysis suggested that certain metabolites were dysregulated across independent animal studies and expanded the understanding of the physiopathology of depression.

In the current study, we found that a variety of neurotransmitters were downregulated in the brains of depression models. Consistent with our expectations, we found that the central levels of monoamine neurotransmitters, including serotonin, dopamine, and norepinephrine, were decreased, supporting the classical monoamine hypothesis of depression [32]. We also found abnormalities in amino acid neurotransmitters, such as decreases in GABA levels in the brain and glutamic acid levels in the prefrontal cortex. These results are in agreement with the findings of previous meta-analyses, which showed lower brain GABA and glutamatergic metabolite levels in MDD patients [33–35]. These findings confirmed the role of altered glutamatergic and GABAergic neurotransmission in the pathophysiology of depression, as described in a previous review [36]. Another noteworthy observation is that we found downregulated anandamide levels in the brain. A relationship between anandamide, one of the main endocannabinoid metabolites, and the emotional state of animals was suggested. Previous studies found that decreasing anandamide levels by depletion of its producing enzyme may be a risk for the development of depression [37], and increasing its levels by blocking its hydrolase exerted antidepressant effects [38].

The results of this study showed upregulated kynurenine and hydroxykynurenine levels, as well as downregulated tryptophan levels in the brain. Kynurenine is synthesized from tryptophan and then broken down into hydroxykynurenine. The increased kynurenine to tryptophan ratio also suggested the activation of tryptophan metabolism to kynurenine by indoleamine 2,3-dioxygenase [39]. Disturbances of kynurenine metabolites are associated with neuroimmune disturbance, as elevated kynurenine induced depression-like behaviors in rodents through monocyte trafficking and regulation of the NLRP2 inflammasome in astrocytes [40, 41]. Therefore, this evidence highlighted that the neurotoxicity of kynurenine is a potential contributor to the pathogenesis of depression. Despite these findings, postmortem studies demonstrated unaltered in brain tissues of depressed individuals [42, 43], and previous meta-analyses reported decreased kynurenine levels and unaltered hydroxykynurenine concentrations in the blood of MDD patients [44, 45]. Therefore, further studies that determining the central levels of the kynurenine metabolites in MDD patients are still needed.

We found that the alterations in the blood of animal models were mainly characterized by decreases of amino acid concentrations. Two branched-chain amino acids (valine and leucine), two aromatic amino acids (tryptophan and tyrosine), and one other amino acid (proline) were downregulated. These results are in line with those of our recent meta-analysis, which showed a trend or statistical significance in decreases in these metabolites in the blood of MDD patients [22]. However, a decrease in the levels of these amino acids was not demonstrated to be a causal factor in the pathogenesis of depression, as depletion of these amino acids did not decrease mood in humans [46, 47]. The possible reason for these findings is that stressed animals were in chronic catabolic states because of reduced food intake and weight loss, which may lead to disturbances in amino acid metabolism. In addition, we also identified upregulated glutamine levels. The association of circulating glutamine levels and depression is still controversial in clinical settings, as our previous meta-analysis reported decreased glutamine levels in MDD patients [22], while unaltered levels were reported in other meta-analyses [48, 49]. Therefore, further research on this topic is still needed.

Our results suggested upregulated corticosterone levels in the blood. Consistent with findings in MDD patients [50], increased circulating corticosterone supported the stress-induced hyperactivity of the hypothalamus–pituitary–adrenal axis, as previously discussed [51]. We also found that N-acetyl glycoprotein was upregulated in the blood, which is consistent with data obtained in human studies [48]. N-Acetyl glycoprotein is a circulating marker of systemic inflammation because its concentration correlates with C-reactive protein, fibrinogen, and interleukin-6 levels [52, 53]. Therefore, these findings support that disturbances in the hypothalamus–pituitary–adrenal axis and peripheral inflammation are involved in the development of depression.

We found that three metabolites (citric acid, oxoglutaric acid, and creatine) involved in recycling of adenosine triphosphate (ATP) were downregulated in the urine of depression models. Moreover, pyruvic acid, an important metabolite in ATP metabolism, was also downregulated in the blood. These altered metabolites suggested an imbalance of energy metabolism in depression, which also could be explained by the chronic catabolic state. It was noted that supplementation of creatine or ATP resulted in antidepressant activity in preclinical studies [54, 55]; therefore, energy metabolism may serve as a potential target for depression treatment.

Among the altered metabolites in urine, we also found that gut microbiota contributed to the physiopathology of depression. Hippuric acid was the only upregulated metabolite in urine, and its production in rats largely depends on the gut microflora [56]. We also identified downregulation of L-dopa, betaine, and tryptophan levels in urine and decreased trimethylamine N-oxide levels in blood. The biochemical transformations of these metabolites involve a variety of gut microbiota, as described in a previous review [57]. Therefore, alterations in these microbial metabolites suggest the involvement of gut microbes in the development of depression, while further studies investigating the underlying pathogenic bacterium are still warranted.

Among the altered metabolites, tryptophan and serotonin were downregulated in both brain and peripheral tissues, and proline was downregulated in blood and urine samples. Moreover, some metabolites showed similar dysregulated trends in central and peripheral tissues. For example, tyrosine is the precursor of the monoamine neurotransmitters dopamine and epinephrine, its concentration was reduced in blood and also showed a downregulation trend in the brain and urine. In addition, it is also noticed an accumulation of kynurenine in the brain of depression models, with an increased trend in the blood. Central kynurenine is mainly synthesized from peripheral tryptophan degradation and then taken up into brain by transporters [58]. In MDD patients, plasma kynurenine levels correlated with its levels in the cerebrospinal fluid [59]. Other studies reported that attenuating the kynurenine accumulation in the brain by lowering plasma kynurenine levels or blocking the entry of kynurenine into the brain can exert an antidepressant effect [60, 61]. Therefore, these findings highlight the bidirectional interactions of metabolomic changes between the brain and peripheral tissues.

Our study presents some limitations. First, application of the vote counting approach cannot identify new differential metabolites other than candidate metabolites. However, because only 3% of the included studies provided raw data, this method is still the most feasible way to perform such a large-scale quantitative analysis. To increase the accessibility of raw data, further studies with greater statistical power should be performed. Second, some metabolites were excluded from the semi-quantitative analysis because of an insufficient number of studies. Moreover, limited metabolite entries precluded stratified analyses. Future studies with more included studies are therefore recommended. Third, the metabolic alterations reported in this study may be caused by stress and are not specific to depression; therefore, further studies are needed to determine the causal relationship between these altered metabolites and the onset of depression. Finally, the publication bias was not examined as in an ordinary meta-analysis because of the use of vote counting.

In conclusion, we performed a quantitative analysis to identify consistently altered metabolites in the brain, blood, and urine of depression models based on 3743 differential metabolites from 241 metabolomics studies. The findings of this study suggested that metabolomic changes in depression models are characterized by decreased neurotransmitter and increased kynurenine metabolite levels in the brain, decreased amino acid and increased corticosterone levels in the blood, and imbalanced energy metabolism and microbial metabolites in urine. This study contributes to the existing knowledge of metabolomic changes in depression and reveals that the reproducibility of candidate metabolites was inadequate in previous studies.

## Supplementary information

Supplementary Table 1

Supplementary Table 2

Supplementary Table 3

Supplementary Table 4

Supplementary Table 5

Supplementary Table 6

Supplementary Table 7

Supplementary Table 8

Supplementary Table 9

Supplementary Table 10

Supplementary Table 11

Supplementary Table 12

Supplementary Data 1

Supplementary Data 2

Supplementary Data 3

Supplementary Data 4

Supplementary Data 5
