# Supplementary material for: Metabolomic changes in animal models of depression: a systematic analysis
Source: Mol Psychiatry. 2021 Sep 1;26(12):7328–36. doi: 10.1038/s41380-021-01269-w (PMC8872989; doi:10.1038/s41380-021-01269-w)
Supplement: Supplementary file 1 — Supplementary Table 1 [file 41380_2021_1269_MOESM1_ESM.docx]

| **Supplementary Table 1. Numbers of studies and differential metabolites excluded from MENDA database.** | | |
| --- | --- | --- |
| **Category** | **No. of excluded studies** | **No. of excluded metabolites** |
| Human study | 342 | 2,428 |
| Non-human primate model study | 8 | 703 |
| Cell model study | 2 | 91 |
| Intervention study | 113 | 4,007 |
| Magnetic resonance spectroscopy study | 24 | 107 |
| Other tissues than brain, plasma, serum, or urine | 38 | 869 |
| Candidate metabolites with unknown regulation direction | 0 | 149 |
| **Total** | **527** | **8,354** |
