# Supplementary material for: Metabolomic changes in animal models of depression: a systematic analysis
Source: Mol Psychiatry. 2021 Sep 1;26(12):7328–36. doi: 10.1038/s41380-021-01269-w (PMC8872989; doi:10.1038/s41380-021-01269-w)
Supplement: Supplementary file 2 — Supplementary Table 2 [file 41380_2021_1269_MOESM2_ESM.docx]

| **Supplementary Table 2. Numbers of studies and differential metabolites in this study.** | | |
| --- | --- | --- |
| **Category** | **No. of studies**  **(n = 241)** | **No. of metabolites**  **(n = 3,743)** |
| Depression model |  |  |
| Chronic mild stress model | 140 | 1,955 |
| Social defeat model | 17 | 568 |
| LPS model | 17 | 240 |
| Chronic restraint stress model | 12 | 153 |
| Learned helplessness model | 2 | 44 |
| Other animal models | 57 | 783 |
| Tissue |  |  |
| Brain | 151 | 2,119 |
| Hippocampus | 95 | 1,119 |
| Prefrontal cortex | 46 | 460 |
| Plasma | 53 | 415 |
| Serum | 43 | 572 |
| Urine | 37 | 637 |
| Organism |  |  |
| Rat | 191 | 2,513 |
| Mouse | 88 | 1,230 |
| Platform |  |  |
| Mass spectrometry | 200 | 3,258 |
| Nuclear magnetic resonance | 49 | 485 |
| *LPS model*, lipopolysaccharide induced depression model. | | |
