# Supplementary material for: Metabolomic changes in animal models of depression: a systematic analysis
Source: Mol Psychiatry. 2021 Sep 1;26(12):7328–36. doi: 10.1038/s41380-021-01269-w (PMC8872989; doi:10.1038/s41380-021-01269-w)
Supplement: Supplementary file 3 — Supplementary Table 3 [file 41380_2021_1269_MOESM3_ESM.docx]

| **Supplementary Table 3. Vote counting results for the brain.** | | | | | |
| --- | --- | --- | --- | --- | --- |
| **Metabolites** | **Vote counting statistic** | **No. of studies that report on the metabolite** | | | ***P* value** |
|  |  | **All** | **Upregulated** | **Downregulated** |  |
| Serotonin | −28 | 42 | 7 | 35 | <0.001 |
| Dopamine | −24 | 34 | 5 | 29 | <0.001 |
| Gamma-Aminobutyric acid | −21 | 51 | 15 | 36 | 0.002 |
| Norepinephrine | −11 | 23 | 6 | 17 | 0.017 |
| N-Acetyl-L-aspartic acid | −11 | 37 | 13 | 24 | 0.049 |
| Anandamide | −10 | 18 | 4 | 14 | 0.015 |
| L-Tryptophan | −10 | 30 | 10 | 20 | 0.049 |
| L-Glutamine | −8 | 40 | 16 | 24 | 0.134 |
| L-Tyrosine | −7 | 15 | 4 | 11 | 0.059 |
| L-Aspartic acid | −6 | 24 | 9 | 15 | 0.154 |
| Docosahexaenoic acid | −5 | 7 | 1 | 6 | 0.063 |
| Niacinamide | −5 | 7 | 1 | 6 | 0.063 |
| Cholesterol | −5 | 11 | 3 | 8 | 0.113 |
| L-Proline | −5 | 11 | 3 | 8 | 0.113 |
| Creatine | −5 | 15 | 5 | 10 | 0.151 |
| Epinephrine | −4 | 4 | 0 | 4 | 0.063 |
| Guanosine | −4 | 4 | 0 | 4 | 0.063 |
| Pantothenic acid | −4 | 4 | 0 | 4 | 0.063 |
| 5-Hydroxytryptophol | −4 | 6 | 1 | 5 | 0.109 |
| Citric acid | −4 | 8 | 2 | 6 | 0.145 |
| Hypoxanthine | −4 | 8 | 2 | 6 | 0.145 |
| L-Dopa | −4 | 8 | 2 | 6 | 0.145 |
| L-Lysine | −4 | 8 | 2 | 6 | 0.145 |
| 5-Hydroxyindoleacetic acid | −4 | 16 | 6 | 10 | 0.227 |
| Taurine | −4 | 18 | 7 | 11 | 0.240 |
| Kynurenic acid | −3 | 9 | 3 | 6 | 0.254 |
| MG(0:0/20:4(5Z,8Z,11Z,14Z)/0:0) | −3 | 13 | 5 | 8 | 0.291 |
| L-Phenylalanine | −3 | 17 | 7 | 10 | 0.315 |
| Adenine | −2 | 4 | 1 | 3 | 0.313 |
| Dihydroxyacetone phosphate | −2 | 4 | 1 | 3 | 0.313 |
| D-Ribose 5-phosphate | −2 | 4 | 1 | 3 | 0.313 |
| Fosfomycin | −2 | 4 | 1 | 3 | 0.313 |
| N-acetyltryptophan | −2 | 4 | 1 | 3 | 0.313 |
| Pipecolic acid | −2 | 4 | 1 | 3 | 0.313 |
| Choline | −2 | 6 | 2 | 4 | 0.344 |
| Pyroglutamic acid | −2 | 8 | 3 | 5 | 0.363 |
| Homovanillic acid | −2 | 10 | 4 | 6 | 0.377 |
| 5-Hydroxy-L-tryptophan | −1 | 5 | 2 | 3 | 0.500 |
| Cyclic AMP | −1 | 5 | 2 | 3 | 0.500 |
| L-Allothreonine | −1 | 5 | 2 | 3 | 0.500 |
| L-Histidine | −1 | 5 | 2 | 3 | 0.500 |
| L-Isoleucine | −1 | 5 | 2 | 3 | 0.500 |
| L-Asparagine | −1 | 7 | 3 | 4 | 0.500 |
| L-Cysteine | −1 | 7 | 3 | 4 | 0.500 |
| 3,4-Dihydroxybenzeneacetic acid | −1 | 9 | 4 | 5 | 0.500 |
| L-Serine | −1 | 9 | 4 | 5 | 0.500 |
| L-Leucine | −1 | 11 | 5 | 6 | 0.500 |
| Inosine | −1 | 17 | 8 | 9 | 0.500 |
| L-Alanine | 0 | 18 | 9 | 9 | 0.593 |
| Acetylcholine | 0 | 12 | 6 | 6 | 0.613 |
| Ethanolamine | 0 | 10 | 5 | 5 | 0.623 |
| L-Methionine | 0 | 8 | 4 | 4 | 0.637 |
| L-Valine | 0 | 8 | 4 | 4 | 0.637 |
| Phosphate | 0 | 8 | 4 | 4 | 0.637 |
| Aminooxyacetic acid | 0 | 6 | 3 | 3 | 0.656 |
| Hexadecane | 0 | 6 | 3 | 3 | 0.656 |
| Palmitic acid | 0 | 6 | 3 | 3 | 0.656 |
| 3-Phosphoglyceric acid | 0 | 4 | 2 | 2 | 0.688 |
| L-Arginine | 0 | 4 | 2 | 2 | 0.688 |
| N-Acetylleucine | 0 | 4 | 2 | 2 | 0.688 |
| Xanthine | 0 | 4 | 2 | 2 | 0.688 |
| Stearic acid | 1 | 7 | 4 | 3 | 0.500 |
| Glycine | 1 | 15 | 8 | 7 | 0.500 |
| Arachidonic acid | 1 | 17 | 9 | 8 | 0.500 |
| Beta-Alanine | 2 | 4 | 3 | 1 | 0.313 |
| Myristic acid | 2 | 4 | 3 | 1 | 0.313 |
| Urea | 2 | 4 | 3 | 1 | 0.313 |
| Ascorbic acid | 2 | 6 | 4 | 2 | 0.344 |
| Glutathione | 2 | 6 | 4 | 2 | 0.344 |
| Vanillylmandelic acid | 2 | 6 | 4 | 2 | 0.344 |
| LysoPC(16:0) | 2 | 8 | 5 | 3 | 0.363 |
| O-Phosphoethanolamine | 2 | 8 | 5 | 3 | 0.363 |
| N-Methylhydantoin | 2 | 10 | 6 | 4 | 0.377 |
| Glycerol | 2 | 12 | 7 | 5 | 0.387 |
| 3-Hydroxyanthranilic acid | 3 | 5 | 4 | 1 | 0.188 |
| Xanthurenic acid | 4 | 4 | 4 | 0 | 0.063 |
| Glycerophosphocholine | 4 | 8 | 6 | 2 | 0.145 |
| L-Threonine | 4 | 10 | 7 | 3 | 0.172 |
| Succinic acid | 4 | 10 | 7 | 3 | 0.172 |
| L-Glutamic acid | 4 | 54 | 29 | 25 | 0.342 |
| 5-HIAA/5-HT ratio | 5 | 7 | 6 | 1 | 0.063 |
| Quinolinic acid | 5 | 7 | 6 | 1 | 0.063 |
| L-Lactic acid | 6 | 20 | 13 | 7 | 0.132 |
| Kynurenine/tryptophan ratio | 8 | 8 | 8 | 0 | 0.004 |
| Hydroxykynurenine | 11 | 13 | 12 | 1 | 0.002 |
| myo-Inositol | 14 | 24 | 19 | 5 | 0.003 |
| L-Kynurenine | 19 | 21 | 20 | 1 | <0.001 |
| *5-HIAA*, 5-hydroxyindoleacetic acid; *5-HT*, serotonin; *AMP*, adenosine monophosphate; *LysoPC*, lysophosphatidylcholine; *MG*, monoacylglycerol. | | | | | |
