# Supplementary material for: Metabolomic changes in animal models of depression: a systematic analysis
Source: Mol Psychiatry. 2021 Sep 1;26(12):7328–36. doi: 10.1038/s41380-021-01269-w (PMC8872989; doi:10.1038/s41380-021-01269-w)
Supplement: Supplementary file 4 — Supplementary Table 4 [file 41380_2021_1269_MOESM4_ESM.docx]

| **Supplementary Table 4. Vote counting results for hippocampus.** | | | | | |
| --- | --- | --- | --- | --- | --- |
| **Metabolites** | **Vote counting statistic** | **No. of studies that report on the metabolite** | | | ***P* value** |
|  |  | **All** | **Upregulated** | **Downregulated** |  |
| Serotonin | −17 | 23 | 3 | 20 | <0.001 |
| Dopamine | −13 | 21 | 4 | 17 | 0.004 |
| Gamma-Aminobutyric acid | −10 | 26 | 8 | 18 | 0.038 |
| L-Tryptophan | −7 | 17 | 5 | 12 | 0.072 |
| N-Acetyl-L-aspartic acid | −5 | 19 | 7 | 12 | 0.180 |
| Anandamide | −4 | 6 | 1 | 5 | 0.109 |
| Cholesterol | −4 | 6 | 1 | 5 | 0.109 |
| Creatine | −4 | 8 | 2 | 6 | 0.145 |
| L-Tyrosine | −4 | 8 | 2 | 6 | 0.145 |
| L-Aspartic acid | −4 | 10 | 3 | 7 | 0.172 |
| L-Phenylalanine | −4 | 10 | 3 | 7 | 0.172 |
| Norepinephrine | −4 | 10 | 3 | 7 | 0.172 |
| MG(0:0/20:4(5Z,8Z,11Z,14Z)/0:0) | −3 | 5 | 1 | 4 | 0.188 |
| Citric acid | −2 | 4 | 1 | 3 | 0.313 |
| Kynurenic acid | −2 | 4 | 1 | 3 | 0.313 |
| Niacinamide | −2 | 4 | 1 | 3 | 0.313 |
| Ethanolamine | −2 | 6 | 2 | 4 | 0.344 |
| Hypoxanthine | −2 | 6 | 2 | 4 | 0.344 |
| Pyroglutamic acid | −2 | 8 | 3 | 5 | 0.363 |
| Taurine | −2 | 8 | 3 | 5 | 0.363 |
| Acetylcholine | −1 | 5 | 2 | 3 | 0.500 |
| Aminooxyacetic acid | −1 | 5 | 2 | 3 | 0.500 |
| L-Valine | −1 | 5 | 2 | 3 | 0.500 |
| Glycine | −1 | 7 | 3 | 4 | 0.500 |
| L-Leucine | −1 | 7 | 3 | 4 | 0.500 |
| L-Serine | −1 | 7 | 3 | 4 | 0.500 |
| Phosphate | −1 | 7 | 3 | 4 | 0.500 |
| 5-Hydroxyindoleacetic acid | −1 | 9 | 4 | 5 | 0.500 |
| L-Glutamine | −1 | 17 | 8 | 9 | 0.500 |
| 3-Phosphoglyceric acid | 0 | 4 | 2 | 2 | 0.688 |
| Ascorbic acid | 0 | 4 | 2 | 2 | 0.688 |
| Glutathione | 0 | 4 | 2 | 2 | 0.688 |
| L-Allothreonine | 0 | 4 | 2 | 2 | 0.688 |
| L-Arginine | 0 | 4 | 2 | 2 | 0.688 |
| Xanthine | 0 | 4 | 2 | 2 | 0.688 |
| Hexadecane | 0 | 6 | 3 | 3 | 0.656 |
| N-Methylhydantoin | 0 | 6 | 3 | 3 | 0.656 |
| Arachidonic acid | 0 | 10 | 5 | 5 | 0.623 |
| 3,4-Dihydroxybenzeneacetic acid | 1 | 5 | 3 | 2 | 0.500 |
| Glycerophosphocholine | 1 | 5 | 3 | 2 | 0.500 |
| Homovanillic acid | 1 | 5 | 3 | 2 | 0.500 |
| L-Methionine | 1 | 5 | 3 | 2 | 0.500 |
| L-Threonine | 1 | 5 | 3 | 2 | 0.500 |
| Glycerol | 1 | 9 | 5 | 4 | 0.500 |
| Myristic acid | 2 | 4 | 3 | 1 | 0.313 |
| LysoPC(16:0) | 2 | 6 | 4 | 2 | 0.344 |
| O-Phosphoethanolamine | 2 | 8 | 5 | 3 | 0.363 |
| Inosine | 2 | 10 | 6 | 4 | 0.377 |
| Quinolinic acid | 3 | 5 | 4 | 1 | 0.188 |
| L-Alanine | 3 | 7 | 5 | 2 | 0.227 |
| L-Lactic acid | 3 | 13 | 8 | 5 | 0.291 |
| Hydroxykynurenine | 4 | 4 | 4 | 0 | 0.063 |
| 5-HIAA/5-HT ratio | 4 | 6 | 5 | 1 | 0.109 |
| Succinic acid | 4 | 6 | 5 | 1 | 0.109 |
| Kynurenine/tryptophan ratio | 5 | 5 | 5 | 0 | 0.031 |
| myo-Inositol | 6 | 14 | 10 | 4 | 0.090 |
| L-Glutamic acid | 8 | 30 | 19 | 11 | 0.100 |
| L-Kynurenine | 11 | 13 | 12 | 1 | 0.002 |
| *5-HIAA*, 5-hydroxyindoleacetic acid; *5-HT*, serotonin; *LysoPC*, lysophosphatidylcholine; *MG*, monoacylglycerol. | | | | | |
