# Supplementary material for: Metabolomic changes in animal models of depression: a systematic analysis
Source: Mol Psychiatry. 2021 Sep 1;26(12):7328–36. doi: 10.1038/s41380-021-01269-w (PMC8872989; doi:10.1038/s41380-021-01269-w)
Supplement: Supplementary file 5 — Supplementary Table 5 [file 41380_2021_1269_MOESM5_ESM.docx]

| **Supplementary Table 5. Vote counting results for prefrontal cortex.** | | | | | |
| --- | --- | --- | --- | --- | --- |
| **Metabolites** | **Vote counting statistic** | **No. of studies that report on the metabolite** | | | ***P* value** |
|  |  | **All** | **Upregulated** | **Downregulated** |  |
| L-Glutamic acid | −8 | 12 | 2 | 10 | 0.019 |
| L-Glutamine | −6 | 12 | 3 | 9 | 0.073 |
| Gamma-Aminobutyric acid | −5 | 9 | 2 | 7 | 0.090 |
| Norepinephrine | −4 | 4 | 0 | 4 | 0.063 |
| Serotonin | −4 | 6 | 1 | 5 | 0.109 |
| Homovanillic acid | −2 | 4 | 1 | 3 | 0.313 |
| L-Aspartic acid | −2 | 4 | 1 | 3 | 0.313 |
| MG(0:0/20:4(5Z,8Z,11Z,14Z)/0:0) | −2 | 4 | 1 | 3 | 0.313 |
| N-acetyltryptophan | −2 | 4 | 1 | 3 | 0.313 |
| Creatine | −2 | 6 | 2 | 4 | 0.344 |
| N-Acetyl-L-aspartic acid | −2 | 10 | 4 | 6 | 0.377 |
| Taurine | −1 | 5 | 2 | 3 | 0.500 |
| Arachidonic acid | 0 | 4 | 2 | 2 | 0.688 |
| L-Alanine | 1 | 5 | 3 | 2 | 0.500 |
| myo-Inositol | 2 | 4 | 3 | 1 | 0.313 |
| *MG*, monoacylglycerol. | | | | | |
