# Supplementary material for: Metabolomic changes in animal models of depression: a systematic analysis
Source: Mol Psychiatry. 2021 Sep 1;26(12):7328–36. doi: 10.1038/s41380-021-01269-w (PMC8872989; doi:10.1038/s41380-021-01269-w)
Supplement: Supplementary file 6 — Supplementary Table 6 [file 41380_2021_1269_MOESM6_ESM.docx]

| **Supplementary Table 6. Vote counting results for blood.** | | | | | |
| --- | --- | --- | --- | --- | --- |
| **Metabolites** | **Vote counting statistic** | **No. of studies that report on the metabolite** | | | ***P* value** |
|  |  | **All** | **Upregulated** | **Downregulated** |  |
| L-Tryptophan | −18 | 18 | 0 | 18 | <0.001 |
| L-Leucine | −10 | 10 | 0 | 10 | 0.001 |
| L-Tyrosine | −9 | 9 | 0 | 9 | 0.002 |
| L-Valine | −9 | 11 | 1 | 10 | 0.006 |
| Trimethylamine N-oxide | −9 | 13 | 2 | 11 | 0.011 |
| L-Proline | −8 | 8 | 0 | 8 | 0.004 |
| Oleamide | −6 | 6 | 0 | 6 | 0.016 |
| Pyruvic acid | −6 | 6 | 0 | 6 | 0.016 |
| L-Isoleucine | −6 | 10 | 2 | 8 | 0.055 |
| Serotonin | −5 | 5 | 0 | 5 | 0.031 |
| Creatine | −5 | 11 | 3 | 8 | 0.113 |
| L-Methionine | −4 | 6 | 1 | 5 | 0.109 |
| L-Glutamic acid | −4 | 16 | 6 | 10 | 0.227 |
| Gamma-Aminobutyric acid | −3 | 5 | 1 | 4 | 0.188 |
| Stearic acid | −3 | 5 | 1 | 4 | 0.188 |
| Palmitic acid | −3 | 7 | 2 | 5 | 0.227 |
| Betaine | −2 | 4 | 1 | 3 | 0.313 |
| Linoleic acid | −2 | 4 | 1 | 3 | 0.313 |
| L-Palmitoylcarnitine | −2 | 4 | 1 | 3 | 0.313 |
| L-Threonine | −2 | 4 | 1 | 3 | 0.313 |
| LysoPC(18:0) | −2 | 4 | 1 | 3 | 0.313 |
| myo-Inositol | −2 | 6 | 2 | 4 | 0.344 |
| 3-Hydroxybutyric acid | −2 | 12 | 5 | 7 | 0.387 |
| Citric acid | −1 | 5 | 2 | 3 | 0.500 |
| Leucine or Isoleucine | −1 | 5 | 2 | 3 | 0.500 |
| D-Glucose | −1 | 9 | 4 | 5 | 0.500 |
| LysoPC(16:0) | −1 | 9 | 4 | 5 | 0.500 |
| L-Phenylalanine | −1 | 13 | 6 | 7 | 0.500 |
| Glycerol | 0 | 4 | 2 | 2 | 0.688 |
| Hippuric acid | 0 | 4 | 2 | 2 | 0.688 |
| L-Serine | 0 | 4 | 2 | 2 | 0.688 |
| LysoPC(15:0) | 0 | 4 | 2 | 2 | 0.688 |
| LysoPC(18:2(9Z,12Z)) | 0 | 4 | 2 | 2 | 0.688 |
| LysoPC(20:3(5Z,8Z,11Z)) | 0 | 4 | 2 | 2 | 0.688 |
| Uric acid | 0 | 4 | 2 | 2 | 0.688 |
| Quinolinic acid | 0 | 6 | 3 | 3 | 0.656 |
| L-Alanine | 0 | 12 | 6 | 6 | 0.613 |
| Arachidonic acid | 1 | 5 | 3 | 2 | 0.500 |
| Acetoacetic acid | 2 | 4 | 3 | 1 | 0.313 |
| Indoxyl sulfate | 2 | 4 | 3 | 1 | 0.313 |
| Kynurenine/tryptophan ratio | 2 | 4 | 3 | 1 | 0.313 |
| Lipid | 2 | 4 | 3 | 1 | 0.313 |
| Sphinganine | 2 | 4 | 3 | 1 | 0.313 |
| Taurine | 2 | 4 | 3 | 1 | 0.313 |
| Beta-D-Glucose | 2 | 6 | 4 | 2 | 0.344 |
| LysoPC(20:4(5Z,8Z,11Z,14Z)) | 2 | 6 | 4 | 2 | 0.344 |
| Succinic acid | 2 | 6 | 4 | 2 | 0.344 |
| Cholic acid | 2 | 10 | 6 | 4 | 0.377 |
| Choline | 2 | 12 | 7 | 5 | 0.387 |
| D-Fructose | 3 | 5 | 4 | 1 | 0.188 |
| L-Kynurenine | 3 | 7 | 5 | 2 | 0.227 |
| Phenylacetylglycine | 4 | 4 | 4 | 0 | 0.063 |
| Alpha-D-Glucose | 4 | 10 | 7 | 3 | 0.172 |
| Glycine | 4 | 10 | 7 | 3 | 0.172 |
| L-Lactic acid | 4 | 16 | 10 | 6 | 0.227 |
| L-Glutamine | 6 | 8 | 7 | 1 | 0.035 |
| Corticosterone | 9 | 9 | 9 | 0 | 0.002 |
| N-acetyl glycoprotein | 11 | 11 | 11 | 0 | <0.001 |
| *LysoPC*, lysophosphatidylcholine. | | | | | |
