# Supplementary material for: Metabolomic changes in animal models of depression: a systematic analysis
Source: Mol Psychiatry. 2021 Sep 1;26(12):7328–36. doi: 10.1038/s41380-021-01269-w (PMC8872989; doi:10.1038/s41380-021-01269-w)
Supplement: Supplementary file 7 — Supplementary Table 7 [file 41380_2021_1269_MOESM7_ESM.docx]

| **Supplementary Table 7. Vote counting results for plasma.** | | | | | |
| --- | --- | --- | --- | --- | --- |
| **Metabolites** | **Vote counting statistic** | **No. of studies that report on the metabolite** | | | ***P* value** |
|  |  | **All** | **Upregulated** | **Downregulated** |  |
| L-Tryptophan | −11 | 11 | 0 | 11 | <0.001 |
| L-Tyrosine | −7 | 7 | 0 | 7 | 0.008 |
| 3-Hydroxybutyric acid | −4 | 6 | 1 | 5 | 0.109 |
| Gamma-Aminobutyric acid | −3 | 5 | 1 | 4 | 0.188 |
| L-Valine | −3 | 5 | 1 | 4 | 0.188 |
| Cholic acid | −1 | 5 | 2 | 3 | 0.500 |
| L-Glutamic acid | 0 | 6 | 3 | 3 | 0.656 |
| Quinolinic acid | 0 | 6 | 3 | 3 | 0.656 |
| Creatine | 0 | 4 | 2 | 2 | 0.688 |
| L-Alanine | 0 | 4 | 2 | 2 | 0.688 |
| Leucine or Isoleucine | 0 | 4 | 2 | 2 | 0.688 |
| LysoPC(16:0) | 0 | 4 | 2 | 2 | 0.688 |
| D-Glucose | 1 | 5 | 3 | 2 | 0.500 |
| L-Kynurenine | 1 | 5 | 3 | 2 | 0.500 |
| L-Lactic acid | 1 | 5 | 3 | 2 | 0.500 |
| L-Phenylalanine | 1 | 5 | 3 | 2 | 0.500 |
| Glycine | 2 | 4 | 3 | 1 | 0.313 |
| L-Glutamine | 3 | 5 | 4 | 1 | 0.188 |
| Corticosterone | 8 | 8 | 8 | 0 | 0.004 |
| *LysoPC*, lysophosphatidylcholine. | | | | | |
