# Supplementary material for: Metabolomic changes in animal models of depression: a systematic analysis
Source: Mol Psychiatry. 2021 Sep 1;26(12):7328–36. doi: 10.1038/s41380-021-01269-w (PMC8872989; doi:10.1038/s41380-021-01269-w)
Supplement: Supplementary file 8 — Supplementary Table 8 [file 41380_2021_1269_MOESM8_ESM.docx]

| **Supplementary Table 8. Vote counting results for serum.** | | | | | |
| --- | --- | --- | --- | --- | --- |
| **Metabolites** | **Vote counting statistic** | **No. of studies that report on the metabolite** | | | ***P* value** |
|  |  | **All** | **Upregulated** | **Downregulated** |  |
| L-Leucine | −8 | 8 | 0 | 8 | 0.004 |
| L-Tryptophan | −7 | 7 | 0 | 7 | 0.008 |
| L-Isoleucine | −7 | 9 | 1 | 8 | 0.020 |
| L-Valine | −6 | 6 | 0 | 6 | 0.016 |
| Trimethylamine N-oxide | −6 | 10 | 2 | 8 | 0.055 |
| L-Proline | −5 | 5 | 0 | 5 | 0.031 |
| Pyruvic acid | −5 | 5 | 0 | 5 | 0.031 |
| Creatine | −5 | 7 | 1 | 6 | 0.063 |
| Oleamide | −4 | 4 | 0 | 4 | 0.063 |
| L-Methionine | −4 | 6 | 1 | 5 | 0.109 |
| L-Glutamic acid | −4 | 10 | 3 | 7 | 0.172 |
| D-Glucose | −2 | 4 | 1 | 3 | 0.313 |
| LysoPC(18:0) | −2 | 4 | 1 | 3 | 0.313 |
| L-Phenylalanine | −2 | 8 | 3 | 5 | 0.363 |
| LysoPC(16:0) | −1 | 5 | 2 | 3 | 0.500 |
| Palmitic acid | −1 | 5 | 2 | 3 | 0.500 |
| Choline | −1 | 9 | 4 | 5 | 0.500 |
| L-Alanine | 0 | 8 | 4 | 4 | 0.637 |
| LysoPC(20:4(5Z,8Z,11Z,14Z)) | 0 | 4 | 2 | 2 | 0.688 |
| Beta-D-Glucose | 2 | 4 | 3 | 1 | 0.313 |
| Indoxyl sulfate | 2 | 4 | 3 | 1 | 0.313 |
| Lipid | 2 | 4 | 3 | 1 | 0.313 |
| Taurine | 2 | 4 | 3 | 1 | 0.313 |
| 3-Hydroxybutyric acid | 2 | 6 | 4 | 2 | 0.344 |
| Glycine | 2 | 6 | 4 | 2 | 0.344 |
| Cholic acid | 3 | 5 | 4 | 1 | 0.188 |
| L-Lactic acid | 3 | 11 | 7 | 4 | 0.274 |
| Alpha-D-Glucose | 5 | 7 | 6 | 1 | 0.063 |
| N-acetyl glycoprotein | 8 | 8 | 8 | 0 | 0.004 |
| *LysoPC*, lysophosphatidylcholine. | | | | | |
