# Supplementary material for: Metabolomic changes in animal models of depression: a systematic analysis
Source: Mol Psychiatry. 2021 Sep 1;26(12):7328–36. doi: 10.1038/s41380-021-01269-w (PMC8872989; doi:10.1038/s41380-021-01269-w)
Supplement: Supplementary file 9 — Supplementary Table 9 [file 41380_2021_1269_MOESM9_ESM.docx]

| **Supplementary Table 9. Vote counting results in urine.** | | | | | |
| --- | --- | --- | --- | --- | --- |
| **Metabolites** | **Vote counting statistic** | **No. of studies that report on the metabolite** | | | ***P* value** |
|  |  | **All** | **Upregulated** | **Downregulated** |  |
| Citric acid | −16 | 20 | 2 | 18 | <0.001 |
| Oxoglutaric acid | −10 | 12 | 1 | 11 | 0.003 |
| L-Proline | −8 | 10 | 1 | 9 | 0.011 |
| Creatine | −6 | 8 | 1 | 7 | 0.035 |
| L-Tryptophan | −6 | 8 | 1 | 7 | 0.035 |
| Betaine | −5 | 5 | 0 | 5 | 0.031 |
| L-Dopa | −5 | 5 | 0 | 5 | 0.031 |
| Palmitic acid | −5 | 5 | 0 | 5 | 0.031 |
| Pimelic acid | −5 | 5 | 0 | 5 | 0.031 |
| Succinic acid | −5 | 11 | 3 | 8 | 0.113 |
| 3-Hydroxyhippuric acid | −4 | 4 | 0 | 4 | 0.063 |
| Ascorbic acid | −4 | 4 | 0 | 4 | 0.063 |
| Caproic acid | −4 | 4 | 0 | 4 | 0.063 |
| Indoxyl sulfate | −4 | 4 | 0 | 4 | 0.063 |
| L-Threonine | −4 | 4 | 0 | 4 | 0.063 |
| L-Valine | −4 | 8 | 2 | 6 | 0.145 |
| L-Alanine | −3 | 5 | 1 | 4 | 0.188 |
| Suberic acid | −3 | 5 | 1 | 4 | 0.188 |
| Acetic acid | −3 | 7 | 2 | 5 | 0.227 |
| Creatinine | −3 | 13 | 5 | 8 | 0.291 |
| Uric acid | −2 | 4 | 1 | 3 | 0.313 |
| 5-Hydroxyindoleacetic acid | −2 | 6 | 2 | 4 | 0.344 |
| L-Isoleucine | −2 | 6 | 2 | 4 | 0.344 |
| L-Serine | −2 | 6 | 2 | 4 | 0.344 |
| Pyruvic acid | −2 | 8 | 3 | 5 | 0.363 |
| L-Glutamic acid | −2 | 12 | 5 | 7 | 0.387 |
| Indoleacetic acid | −1 | 5 | 2 | 3 | 0.500 |
| L-Tyrosine | −1 | 9 | 4 | 5 | 0.500 |
| Kynurenic acid | 0 | 12 | 6 | 6 | 0.613 |
| Phenylacetylglycine | 0 | 10 | 5 | 5 | 0.623 |
| Acetoacetic acid | 0 | 4 | 2 | 2 | 0.688 |
| Cyclic AMP | 0 | 4 | 2 | 2 | 0.688 |
| L-Asparagine | 0 | 4 | 2 | 2 | 0.688 |
| L-Kynurenine | 0 | 4 | 2 | 2 | 0.688 |
| L-Lactic acid | 0 | 4 | 2 | 2 | 0.688 |
| L-Leucine | 0 | 4 | 2 | 2 | 0.688 |
| Putrescine | 1 | 5 | 3 | 2 | 0.500 |
| Xanthurenic acid | 1 | 9 | 5 | 4 | 0.500 |
| Allantoin | 2 | 4 | 3 | 1 | 0.313 |
| Taurine | 2 | 8 | 5 | 3 | 0.363 |
| Glycine | 2 | 10 | 6 | 4 | 0.377 |
| L-Glutamine | 2 | 12 | 7 | 5 | 0.387 |
| Tyramine | 3 | 5 | 4 | 1 | 0.188 |
| L-Phenylalanine | 3 | 11 | 7 | 4 | 0.274 |
| Hippuric acid | 10 | 18 | 14 | 4 | 0.015 |
| *AMP*, adenosine monophosphate. | | | | | |
