# Supplementary material for: Metabolomic changes in animal models of depression: a systematic analysis
Source: Mol Psychiatry. 2021 Sep 1;26(12):7328–36. doi: 10.1038/s41380-021-01269-w (PMC8872989; doi:10.1038/s41380-021-01269-w)
Supplement: Supplementary file 10 — Supplementary Table 10 [file 41380_2021_1269_MOESM10_ESM.docx]

| **Supplementary Table 10. Vote counting results in the brain of the chronic mild stress model.** | | | | | |
| --- | --- | --- | --- | --- | --- |
| **Metabolites** | **Vote counting statistic** | **No. of studies that report on the metabolite** | | | ***P* value** |
|  |  | **All** | **Upregulated** | **Downregulated** |  |
| Dopamine | −20 | 26 | 3 | 23 | <0.001 |
| Serotonin | −20 | 28 | 4 | 24 | <0.001 |
| Gamma-Aminobutyric acid | −17 | 33 | 8 | 25 | 0.002 |
| L-Tryptophan | −8 | 16 | 4 | 12 | 0.038 |
| N-Acetyl-L-aspartic acid | −8 | 20 | 6 | 14 | 0.058 |
| Norepinephrine | −7 | 17 | 5 | 12 | 0.072 |
| Anandamide | −6 | 6 | 0 | 6 | 0.016 |
| Cholesterol | −5 | 7 | 1 | 6 | 0.063 |
| L-Tyrosine | −5 | 9 | 2 | 7 | 0.090 |
| L-Glutamine | −5 | 21 | 8 | 13 | 0.192 |
| 5-Hydroxytryptophol | −4 | 6 | 1 | 5 | 0.109 |
| Docosahexaenoic acid | −4 | 6 | 1 | 5 | 0.109 |
| Creatine | −4 | 8 | 2 | 6 | 0.145 |
| L-Aspartic acid | −4 | 12 | 4 | 8 | 0.194 |
| Kynurenic acid | −3 | 5 | 1 | 4 | 0.188 |
| L-Lysine | −3 | 5 | 1 | 4 | 0.188 |
| L-Alanine | −2 | 6 | 2 | 4 | 0.344 |
| 3,4-Dihydroxybenzeneacetic acid | −2 | 8 | 3 | 5 | 0.363 |
| Homovanillic acid | −2 | 8 | 3 | 5 | 0.363 |
| 5-Hydroxyindoleacetic acid | −2 | 10 | 4 | 6 | 0.377 |
| Glycine | −2 | 10 | 4 | 6 | 0.377 |
| Arachidonic acid | −1 | 5 | 2 | 3 | 0.500 |
| L-Isoleucine | −1 | 5 | 2 | 3 | 0.500 |
| L-Proline | −1 | 5 | 2 | 3 | 0.500 |
| Glycerol | −1 | 7 | 3 | 4 | 0.500 |
| Acetylcholine | 0 | 10 | 5 | 5 | 0.623 |
| Glutathione | 0 | 4 | 2 | 2 | 0.688 |
| L-Methionine | 0 | 4 | 2 | 2 | 0.688 |
| Stearic acid | 0 | 4 | 2 | 2 | 0.688 |
| MG(0:0/20:4(5Z,8Z,11Z,14Z)/0:0) | 0 | 6 | 3 | 3 | 0.656 |
| Succinic acid | 1 | 5 | 3 | 2 | 0.500 |
| L-Leucine | 1 | 7 | 4 | 3 | 0.500 |
| Taurine | 1 | 9 | 5 | 4 | 0.500 |
| L-Lactic acid | 1 | 13 | 7 | 6 | 0.500 |
| L-Phenylalanine | 1 | 13 | 7 | 6 | 0.500 |
| Hydroxykynurenine | 2 | 4 | 3 | 1 | 0.313 |
| L-Cysteine | 2 | 4 | 3 | 1 | 0.313 |
| L-Valine | 2 | 6 | 4 | 2 | 0.344 |
| Inosine | 2 | 8 | 5 | 3 | 0.363 |
| L-Glutamic acid | 3 | 29 | 16 | 13 | 0.356 |
| L-Threonine | 4 | 4 | 4 | 0 | 0.063 |
| LysoPC(16:0) | 4 | 6 | 5 | 1 | 0.109 |
| myo-Inositol | 4 | 10 | 7 | 3 | 0.172 |
| L-Kynurenine | 10 | 10 | 10 | 0 | 0.001 |
| *LysoPC*, lysophosphatidylcholine; *MG*, monoacylglycerol. | | | | | |
