# Supplementary material for: Metabolomic changes in animal models of depression: a systematic analysis
Source: Mol Psychiatry. 2021 Sep 1;26(12):7328–36. doi: 10.1038/s41380-021-01269-w (PMC8872989; doi:10.1038/s41380-021-01269-w)
Supplement: Supplementary file 12 — Supplementary Table 12 [file 41380_2021_1269_MOESM12_ESM.docx]

| **Supplementary Table 12. Vote counting results in urine of the chronic mild stress model.** | | | | | |
| --- | --- | --- | --- | --- | --- |
| **Metabolites** | **Vote counting statistic** | **No. of studies that report on the metabolite** | | | ***P* value** |
|  |  | **All** | **Upregulated** | **Downregulated** |  |
| Oxoglutaric acid | −8 | 8 | 0 | 8 | 0.004 |
| Citric acid | −8 | 12 | 2 | 10 | 0.019 |
| Palmitic acid | −5 | 5 | 0 | 5 | 0.031 |
| Pimelic acid | −5 | 5 | 0 | 5 | 0.031 |
| L-Proline | −5 | 7 | 1 | 6 | 0.063 |
| L-Tryptophan | −5 | 7 | 1 | 6 | 0.063 |
| L-Valine | −5 | 7 | 1 | 6 | 0.063 |
| 5-Hydroxyindoleacetic acid | −4 | 4 | 0 | 4 | 0.063 |
| Creatinine | −4 | 6 | 1 | 5 | 0.109 |
| Succinic acid | −4 | 8 | 2 | 6 | 0.145 |
| Creatine | −3 | 5 | 1 | 4 | 0.188 |
| Acetic acid | −2 | 4 | 1 | 3 | 0.313 |
| L-Isoleucine | −2 | 4 | 1 | 3 | 0.313 |
| Indoleacetic acid | −1 | 5 | 2 | 3 | 0.500 |
| L-Serine | −1 | 5 | 2 | 3 | 0.500 |
| Pyruvic acid | −1 | 7 | 3 | 4 | 0.500 |
| L-Glutamic acid | 0 | 10 | 5 | 5 | 0.623 |
| L-Tyrosine | 0 | 8 | 4 | 4 | 0.637 |
| Xanthurenic acid | 0 | 6 | 3 | 3 | 0.656 |
| Acetoacetic acid | 0 | 4 | 2 | 2 | 0.688 |
| L-Kynurenine | 0 | 4 | 2 | 2 | 0.688 |
| Phenylacetylglycine | 1 | 5 | 3 | 2 | 0.500 |
| Kynurenic acid | 1 | 7 | 4 | 3 | 0.500 |
| L-Phenylalanine | 2 | 10 | 6 | 4 | 0.377 |
| Tyramine | 3 | 5 | 4 | 1 | 0.188 |
| Glycine | 3 | 7 | 5 | 2 | 0.227 |
| L-Glutamine | 5 | 9 | 7 | 2 | 0.090 |
| Hippuric acid | 7 | 11 | 9 | 2 | 0.033 |
